# Low-intensity continuous ultrasound to inhibit cancer cell migration

**DOI:** 10.3389/fcell.2022.842965

**Published:** 2023-01-12

**Authors:** Itziar González, Jon Luzuriaga, Alba Valdivieso, Manuel Candil, Jesús Frutos, Jaime López, Luis Hernández, Luis Rodríguez-Lorenzo, Virginia Yagüe, Jose Luis Blanco, Alberto Pinto, Julie Earl

**Affiliations:** ^1^ Group of Ultrasonic Resonators RESULT, Institute of Physical Technologies and Informacion, Consejo Superior de Investigaciones Científicas (CSIC), Madrid, Spain; ^2^ Signaling Lab, Department of Cell Biology and Histology, Faculty of Medicine and Nursing, University of the Basque Country (UPV/EHU), Leioa, Spain; ^3^ Molecular Epidemiology and Predictive Tumor Markers Group, Ramón y Cajal Health Research Institute (IRYCIS), Madrid, Spain; ^4^ Biomedical Research Network in Cancer (CIBERONC), Madrid, Spain; ^5^ Institute of Science and Technology of Polymers ICTP, CSIC, Madrid, Spain; ^6^ Universidad Politécnica de Madrid UPM, Escuela Técnica Superior de Ingenieros de Telecomunicación, Madrid, Spain

**Keywords:** low-intensity ultrasounds, cancer cells, migration, inhibition, tumor, long-term effects

## Abstract

In recent years, it has been verified that collective cell migration is a fundamental step in tumor spreading and metastatic processes. In this paper, we demonstrate for the first time how low-intensity ultrasound produces long-term inhibition of collective migration of epithelial cancer cells in wound healing processes. In particular, we show how pancreatic tumor cells, PANC-1, grown as monolayers *in vitro* respond to these waves at frequencies close to 1 MHz and low intensities (<100 mW cm^−2^) for 48–72 h of culture after some minutes of a single ultrasound irradiation. This new strategy opens a new line of action to block the spread of malignant cells in cancer processes. Despite relevant spatial variations of the acoustic pressure amplitude induced in the assay, the cells behave as a whole, showing a collective dynamic response to acoustic performance. Experiments carried out with samples without previous starving showed remarkable effects of the LICUs from the first hours of culture, more prominent than those with experiments with monolayers subjected to fasting prior to the experiments. This new strategy to control cell migration demonstrating the effectiveness of LICUS on not starved cells opens a new line of action to study effects of *in vivo* ultrasonic actuation on tumor tissues with malignant cells. This is a proof-of-concept study to demonstrate the physical effects of ultrasound stimulation on tumor cell migration. An in-depth biological study of the effects of ultrasounds and underlying biological mechanisms is on-going but out of the scope of this article.

## 1 Introduction

Collective cell migration plays a crucial role in pathological processes such as cancer invasion and metastasis, phenomena that depend on the invasive and migratory capacity of malignant cells (Friedl and Gilmour, 2009; Yilmaz and Christofori, 2010; Li et al., 2013; Haeger et al., 2015). During collective migration, cells move as a group maintaining cell-cell junctions, except for some leader cells at the front, which drive migration by scanning the environment to identify a path, while follower cells contribute to optimizing collective movement (Khalil and Friedl, 2010; Reffay et al., 2011). Microfluidic platforms have made it possible to observe and analyze these processes of cell migration through tissues. *In vitro* experiments show that epithelial leader cells at the edge of a wound undergo longer elongations than other cells on the front edge of the epithelial monolayer that propagate perpendicular to the direction of migration (Wirtz et al., 2011; Vedula et al., 2012; Brugues et al., 2014; Reffay et al., 2014; Yamaguchi et al., 2015; Trepat and Sahai, 2018). The mechanical role of leader cells during collective cell migration and some roles of mechanical forces and cell coupling in collective cell rearrangement and migration *in vitro* systems have been recently analyzed (Trepat et al., 2009; Brugues et al., 2014; Yamaguchi et al., 2015; Trepat and Sahai, 2018).

Traction force measurements have been used to analyze epithelial wound closure in some studies in the literature13. While a wound closes, cells exhibit two types of traction force: forces pointing away from the gap, as classically observed during cell migration, and forces pointing toward the gap. Interestingly, these forces can be attributed to lamellipodial protrusions and actomyosin cable contraction. At late stages of wound closure, traction forces mostly point toward the gap. Analyses of these forces within the migratory cell monolayers show accumulation of intercellular stress from the edge to the inside of the monolayer, which induces a greater tension in the cell-cell junctions (Serra-Picamal et al., 2012; Deforet et al., 2014). Thus, migratory cell monolayers can be seen as tissues under tension.

Other recent studies have shown surprising oscillatory movements of epithelial cells in monolayers (Kocgozlu et al., 2016; Notbohm et al., 2016; Ladoux and Mège, 2017). These oscillations define movements of the cells toward the exterior of the epithelial monolayer, which alternate with movements toward the interior in the radial direction. The unexpected variety of cell movement is related to the complex mechanical behavior of biological tissues (cells can deform) and their active nature nature (Park et al., 2016) (cells can modify their contractility or adhesive properties).

On the other hand, cell density is also an important regulator of collective cell dynamics (Sadati et al., 2013; Garcia et al., 2015; Garg et al., 2019). The average cell velocity stabilizes when confluence is reached and decreases when cell density increases, leading to a switch from a fluid-like to a more solid-like state (Sadati et al., 2013). As cell density increases, each cell within the population becomes increasingly trapped by its neighbors, leading to reduced motion and greater correlation of motion (larger clusters of cells that migrate together). This is known as the “cell jamming” effect (Cios et al., 2021). The large-scale coordination is also reduced with a rise in cell density, associated with shorter cell displacements (Bazou et al., 2017; Suarez-Arnedo et al., 2020).

Recent literature has shown that collective cell behaviors can be altered by the application of external forces, including gravity (Park et al., 2016) and chemical and physical stimuli3. The inhibitory effects of electromagnetic fields on cell monolayers have been recently analyzed (Health Effects of Exposure to Ultrasound and Infrasound, 2010; Bazou et al., 2017; Suarez-Arnedo et al., 2020). However, the effects generated by oscillatory mechanical forces applied to cancer cell monolayers have not yet been analyzed in the collective migration processes of tumor cells and are discussed in this paper.

Herein, we demonstrate how low-intensity ultrasound produces long-term inhibition of collective migration of epithelial cancer cells, in particular, in cell monolayers at frequencies close to 1 MHz and low intensities (<100 mW cm^−2^) for 48–72 h of culture after some minutes of a single ultrasound irradiation.

## 2 Methods and materials

Conventional tissue culture assays, ultrasound generators, incubators with CO_2_ supply and imaging equipment have been used for experiments on cell monolayers actuated by ultrasonic transducers. Scratched monolayers of the confluent pancreatic cell line Panc-1 were selected as samples to analyze wound healing processes with and without previous ultrasound irradiation.

### 2.1 Experimental setup

Conventional 8-well plates with rectangular geometry and flat bottom are ultrasonically driven from a piezoelectric ceramic PZ26 (Ferroperm) of a rectangular area (30 mm × 15 mm x 1.5 mm). A 10 V peak-to-peak electrical signal is supplied to the transducer at a frequency of 1 MHz, generating a continuous sine wave. The transducer is attached underneath one well containing the scratched monolayer for the transmission of vibrations.

**FIGURE 1:**
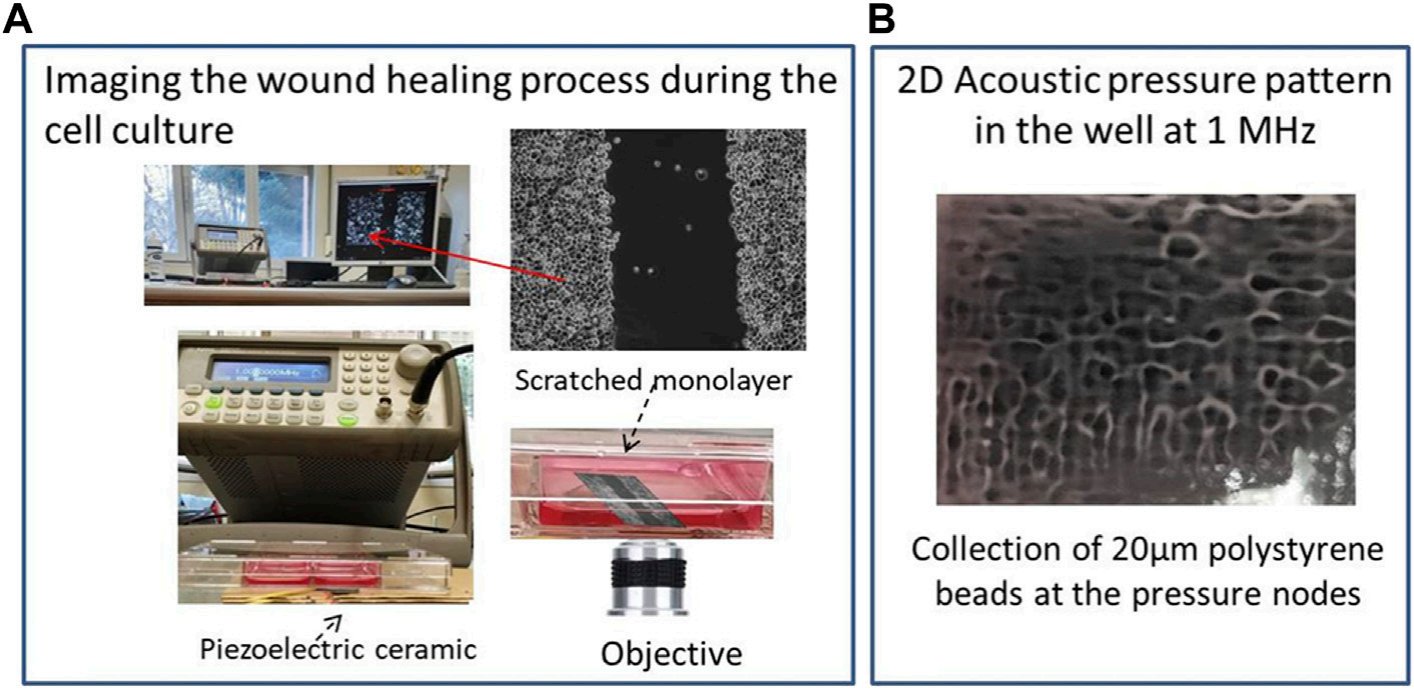
**(A)** Experimental setup including the ultrasound generator, transducer, microscope inside the incubator and image processor; **(B)** Acoustic pressure pattern established at f = 1.003 MHz in the well directly located over the ultrasonic actuator. Polystyrene beads (20 µm) collect at the pressure nodes, drawing this pattern.

The imaging equipment consists of a bright-field microscope (Smart Cell Imager Paula of Leica Microsystems) equipped with a digital camera running its specific software for the acquisition and control of images/video during the entire culture process (Figure 1A). Thus, the dynamics of the cells are monitored in real time after their exposure to the LICU every 10 min, for long periods of up to 72 h of culture, depending on the stage of the wound healing process filmed by the microscope. NIH Science ImageJ freeware was used to process the filmed images, allowing reconstruction of the single-cell trajectories (https://image.net/software/imagej/, University of Wisconsin-Madison).

Figure 1A shows the experimental setup that includes an incubator with a microscope inside controlled by a external computer, an ultrasonic transducer, a signal generator and well plate including the cell samples exposed to the ultrasounds.

### 2.2 Experimental procedure

(i) Cell culture preparation, (ii) scratch-making, (iii) wound healing assays: data acquisition and determination of wound healing rates, (iv) application of ultrasounds and wound healing assays, and (v) data analysis.

#### 2.2.1 Cell culture preparation

The PANC-1 is an epithelioid carcinoma cell line derived from the human pancreas (Deer et al., 2010) and forms part of the ATCC human cell culture collection (https://www.atcc.org/). Standard cell culture protocols for PANC-1 cell lines were applied for cell sample preparation following the Cell Growth Protocol for PANC­1 cell line, 2009. PANC-1 cell lines were cultured in RPMI (Gibco/Invitrogen) supplemented with 10% fetal bovine serum (FBS; Invitrogen) and 50 units/mL penicillin/streptomycin (Invitrogen) and kept in an incubator at 5% CO_2_ and 37°C. Approximately 8.8 × 106 PANC-1 cells were plated in 8-well plates and cultured until confluent monolayers were formed. Then, the cells were either starved in serum-reduced medium (1% serum) for 24 h (starving condition) or maintained in common complete medium (10% serum) (control). During the wound-healing experiments, the cells were exposed to TGF-β stimulation by the addition of 1 µL of recombinant TGF-β to promote cell movement *in vitro.* All methods described were carried out in accordance with relevant guidelines and regulations of the host institutions. Samples from human subjects were not used in this study.

#### 2.2.2 Scratch-Making

The scratches were made by using 2-µL pipette tips with a diameter of approximately 400 microns. Once the cell monolayer is scratched, the wound presents sharp straight boundaries that become blurred as the healing process progresses. Thus, the healing process occurs irregularly. The rate of cell migration can be quantified using two single metrics: the *wound width* as the average distance between the edges of the scratch and/or the *wound area*, which is calculated by the cell-free area in captured images. During this process, nearby leader and follower cells migrate in different directions, not just in the direction of the width of the wound. The cells disperse, making the contours of the wound less sharp and less straight.

#### 2.2.3 Wound healing assays

Data acquisition and determination of wound healing rates Procedure to determine wound healing rates. During the wound-healing experiments, the cells were exposed to TGF-β stimulation by the addition of 1 µL of recombinant TGF-β to promote cell movement *in vitro*. The wounds were photographed at regular intervals every 10 min for long periods of 24, 48 or 72 h. The Alamar Blue assay was used to measure cell viability after each experiment according to the manufacturer’s instructions.

Wound healing rates were also computed from the wound areas measured on filmed images. The wound coverage of the total area and the average and standard deviation of the scratch width was analyzed with the aid of the ImageJ plugin: Wound_healing_size_tool (Pajic-Lijakovic and MIlivojevic, 2020) (WHST). The results of assays were obtained from at least three independent experiments. Migration rate can be expressed as the percentage of area reduction or wound closure from the filmed images during complete wound healing processes (Cukjati et al., 2001). We calculated the rate of cell relative wound closure according to Eq. (1). (Moyano et al., 2022):

$$Wound\;Closure\;\%=\frac{A_{t=0}-A_{t=\Delta h}}{A_{t=0}}\;x\;100$$ (1)

where *A*_*t=0h*_ is the area of the wound measured immediately after scratching (t = 0h) and *A*_*t=𝚫h*_ is the area of the wound measured 𝚫h hours after the scrath performance. The closure percentage will increase as cells migrate over time.

#### 2.2.4 Application of ultrasounds and wound healing assays

The cell samples were exposed to ultrasounds from a piezoelectric actuator attached underneath to the well containing the sample. It was exposed only once per experiment to acoustic intensity levels close to 60 mWatt/cm^2^, significantly lower than a tissue injury threshold described in the literature (Saraswathibhatla et al., 2020) during a time of either 10 min, 15min or 20 min in different experiments immediately before the samples were introduced into the incubator for their culture.

After application of LICUs on the monolayers, the samples were cultured for 2 or 3 days and filmed every 2 hours. LICUs were applied instead of LIPUS (pulsed waves) to control the exact time of action of the monochromatic wave at 1 MHz on the samples, that is, the exact number of acoustic cycles at said frequency. The use of pulsed wave entails a short transitory process until reaching the frequency of the desired wave. This uncertainty time is very short for each wave train, but it becomes relevant throughout the entire ultrasonic actuation of 20 min, with millions of pulses involved.

Figure 1B shows the spatial pressure pattern established at a frequency of 1.003 MHz in a well selected for the experiments, located directly above the ultrasonic actuator. It shows bright areas that correspond to pressure nodes and dark zones to maximum pressure amplitudes of 0.29 MPa measured with a needle hydrophone (Precision Acoustics LTD., hydrophone SN 1423). Polystyrene beads with diameters of 20 µm were used to observe the 2D pressure pattern as they collected at the pressure nodes of the standing waves generated inside the well once driven by a radiation force acoustically induced in the chamber. Nodes and antinodes are separated short distances of less than 1 mm. The wound made in the cell monolayer also includes multiple pressure nodes along its length in both directions, width and length of the gap.

After exposure to different time intervals of 10, 15 or 20 min of ultrasonic treatment, the culture samples were filmed for 48 or 72 h, depending on the closure situation achieved in the wound healing process after the different doses of ultrasonic irradiation.

#### 2.2.5 Data analysis

Statistical Analysis. All results are presented as the mean ± standard error mean (SEM). The Mann-Whitney *U* test was performed to analyze non-parametric results. Statistical tests were performed using SPSS Statistics v.22 (IBM). Statistical significance was considered to be *p ≤ 0.05, **p ≤ 0.01. Complete analyses of cell movements/velocities were mapped and analyzed from a particle imaging velocimetry PIV study running MATLAB, providing velocity fields of the multicell assemblies and individual cells during wound closure, including leaders exploring the substrate and follower cells.

## 3 Results and discussion

Cells normally migrate when exposed to an empty space. Under normal conditions, a wound in monolayers of pancreatic cancer cells takes a little over 24 h to close. These cells have little mobility, so they require longer times than other cell populations.

**FIGURE 2:**
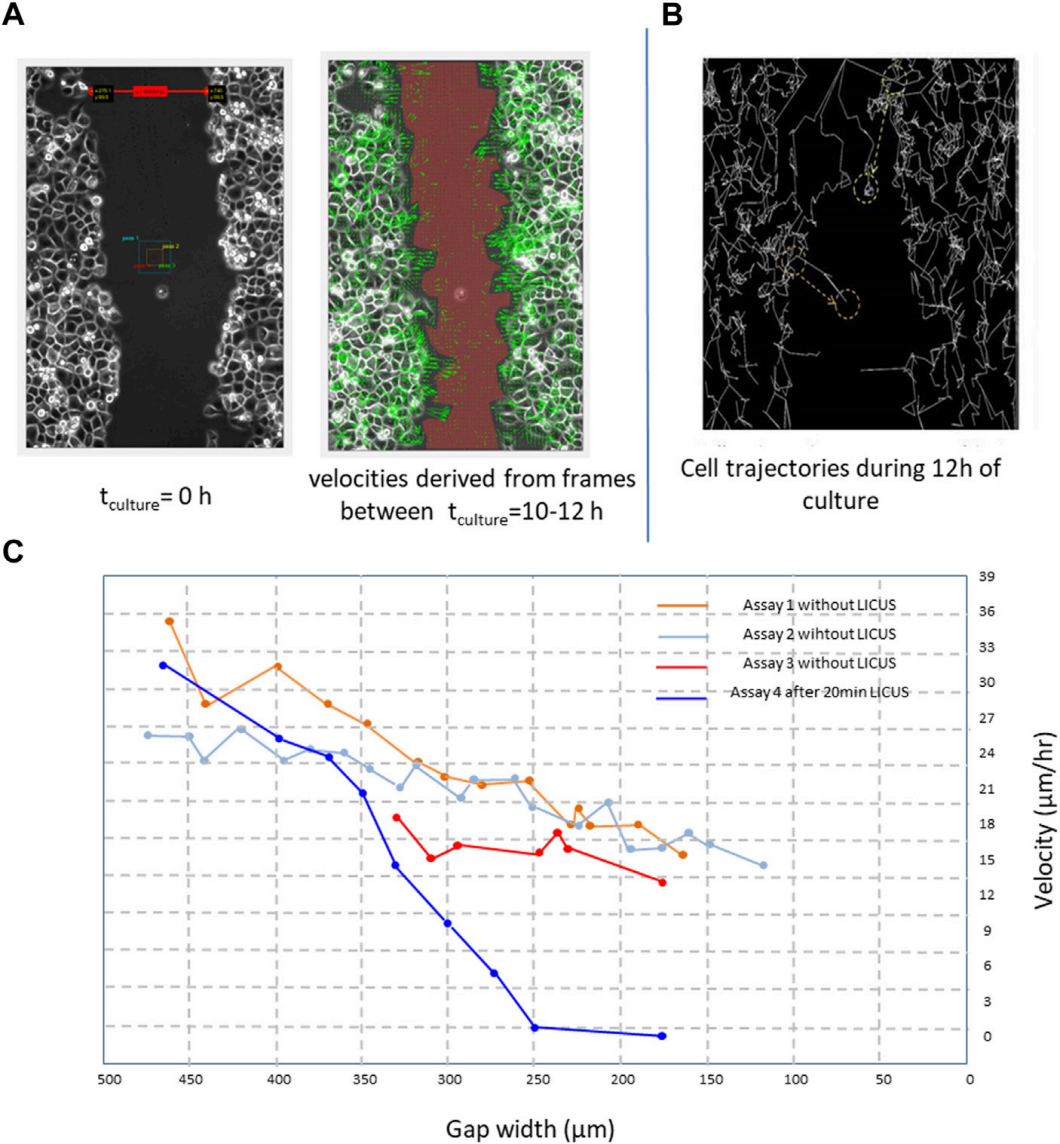
**(A)** PANC-1 cell velocities derived from filmed cell trajectories by PIV-MATLAB code (green arrows) between 4 and 6 h of culture and overimposed on the microscopic image; **(B)** cells trajectories reconstructed in ImageJ freeware (Multitracker plugin) from elapsed frames of a movie during a time of 12 h in a wound closure process; **(C)** derived healing velocity amplitudes measured in five healing processes starting from different wound widths; Scale bar: 100 µm.

Cell movements occur at low Reynolds numbers (Stokes regime), which means that viscous drag of their surrounding medium dominates inertial movements, slowing down the cell movements. In this way, cell dynamics could be assimilated to fluid dynamics. However, cell behaviors cannot be attributed to simple laminar flows. In contrast, cells often show coordinated rotational motions that span over dozens of cells near the edges of the wound in the monolayer, including swirling movements of cell groups and vortices, also observed in these processes (rotation motion is also observable by change of direction of consecutive green arrows in Figure 2B) (Deforet et al., 2014).

### 3.1 Effects of LICUs on cell migration velocities in PANC-1 monolayers

The effects of the LICUS on the progression of the wound healing process were observable since the first hours in the experiments. In each experiment two parameters, wound width and wound area, were respectively analysed for different objectives. The first one was required by MATLAB-PIV software to calculate a mean approach velocity: time-variations in the positions of the individual cells between different filmed frames of a movie, shown in Figure 2A by green arrows. On the other, wound areas were necessary to determine the wound healing rates over time.

The effects of the LICUS on the progression of speed of the cells and wound area were observed from the beginning of the wound healing process, with a tendency to decrease greater than that of a process under normal conditions. Figure 2A shows average velocities of dozens of cells close to both sides of the wound boundary in a time interval ranging from *t*_*1*_ = 15 h and *t*_*2*_ = 20 h after 20min of acoustic treatment in a movie. They are drawn by green arrows over-imposed to a frame of the movie. Leader cells at the edge of the wound describe longer velocity vectors that point in their direction of migration through the substrate or cell gap.

Figure 2B shows trajectories described by hundreds of Panc-1 cells at both sides of a wound scratched in a monolayer 28 h after the ultrasonic irradiation at f∼ 1 MHz. These trajectories were reconstructed in ImageJ freeware from different cells of the monolayer near the wound boundaries after 20 min irradiation of LICUs before culture. During the wound healing process, leader cells enlarge their shapes and acquire higher velocity amplitudes than follower cells to scan the substrate free of cells, according to the literature. However, after LICU irradiation, the leader cells acquire different unexpected behaviors, as described in the following. The leading cells at the border of the migrating tissue adhere and migrate in amaeboid motion on the substrate through the adhesion assembly and extensions of fillopodia that propagate perpendicular to the direction of migration, as described previously in the literature (Wirtz et al., 2011; Brugues et al., 2014; Reffay et al., 2014; Yamaguchi et al., 2015; Trepat and Sahai, 2018).

Figure 2C shows the effects of LICUS on quantified cell velocities in four assays, where the blue graphic corresponding to an assay with previous irradiation of 20min-LICUS decays over time much faster than the other curves, obtained from assays without previous ultrasonic irradiation.

Long-term effects were observed on the cell motion during the whole culture time, as shown in Figure 2C (blue graphic) with quantified cell velocities obtained from four assays. The cells did not perform any individual or collective movements during sonication or during the following 2 hours. In our more than 20 experiments, the cells did not show any dynamics of immediate response to the application of mechanical forces derived from the established acoustic pressure gradient in the well. This shows that the application of LICUS does not produce a Newtonian behavior on the cells, with immediate action/reaction mechanisms. On the contrary, long-term cell alterations on the cell dynamics were found several hours after the LICUS irradiation for at least 2 days of culture after LICUS treatment, depending on the acoustic doses.

**FIGURE 3:**
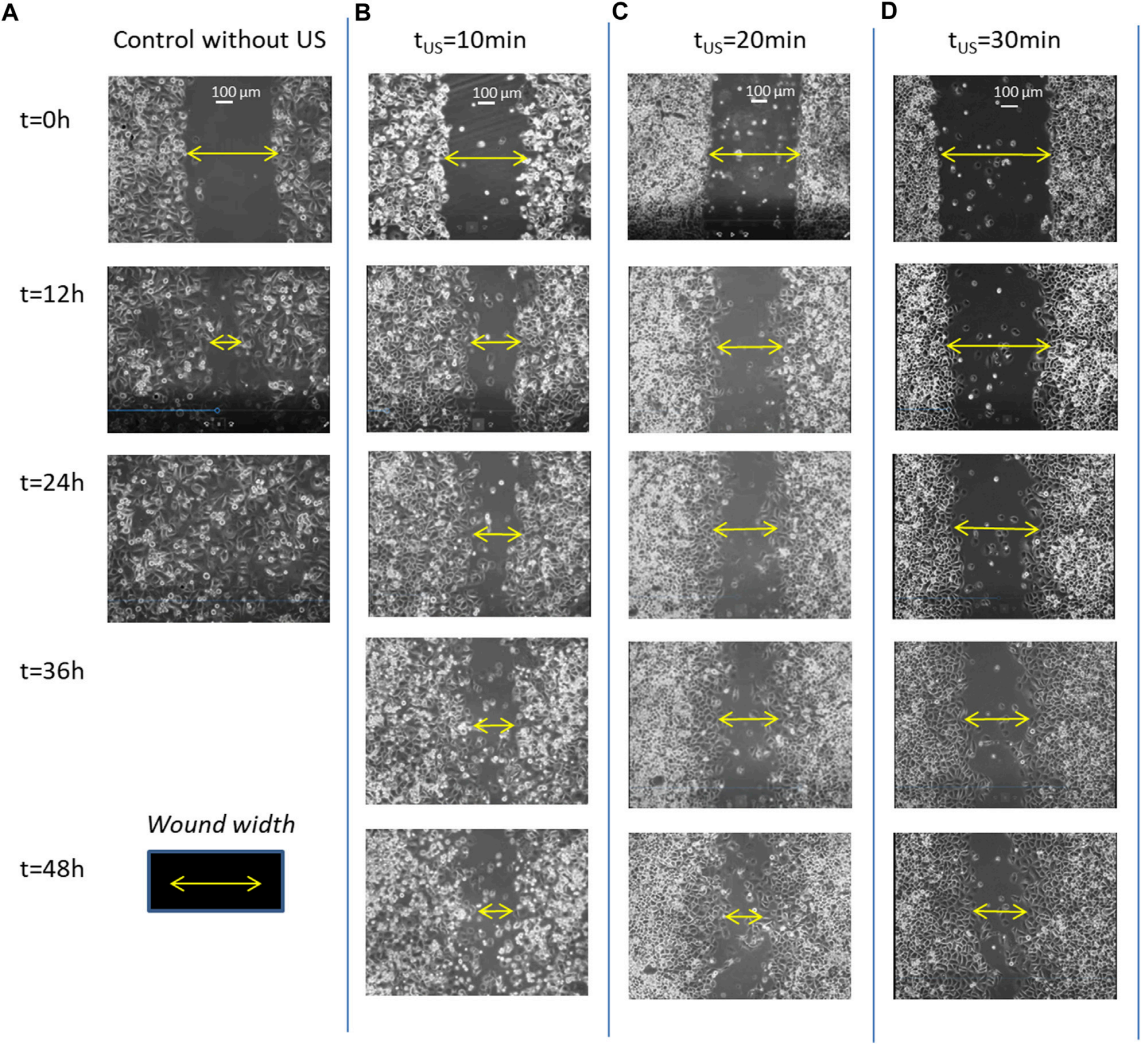
Filmed PANC-1 cell migration during wound healing processes: **(A)** under normal conditions, without ultrasound irradiation; **(B)** after exposure to LICUs at t_US_ = 10 min; **(C)** after LICU irradiation at t_US_ = 20 min; **(D)** t_US_ = 30min before the culture.

Figure 3 shows this long delay effect of the ultrasonic irradiation on the wound healing progression of PANC-1 cell monolayers on three culture processes previously exposed to different doses of ultrasonic irradiation, of 10min, 20min and 30min–LICUS respectively (Figures 3B–D), compared to a normal closure process (Figure 3A). Increasing delays in the wound closure were observed with the longer times of ultrasound irradiation. This inhibitory effect of LICUS induced on cell migration was repeatedly observed in all the 20 experiments.

Supplementary Video S1 shows comparatively two movies taken from wound healing processes experienced by PANC-1 cell monolayers during 48 h: the upper movie corresponds to a sample previously exposed to 20 min of ultrasonic irradiation and lower movie to a wound healing developed under normal conditions (without ultrasound exposure) respectively. A delay of gap closure is observed after acoustic treatment, showing inhibition of collective cell migration. The video includes superimposed colours assigned to each cell quantifying their individual displacements, including amplitude and direction of motion. Red colour refers to right-direction motion, green refers to left-direction, blue one refers to vertical displacements and yellow to stagnant conditions. Several leader and follower cells behind them experience complex reversible and rotational motions over time, showing different colors in the images. Thus, in a natural wound healing process (without any external force applied), PANC-1 leader and follower cells in the first 6-8 rows behind the edge show large displacements toward the wound gap to find cells coming from the faced edge of the gap. Both sets of faced cells confluence in relatively short times close to 1 day. In contrast, the cancer cells in monolayers previously exposed to 20min-LICUs paralyze their migration in time, showing very slow displacements during 48 or even 72 h of culture.

In addition to this effect, we found cancer cell monolayers behaving as single bodies rather than linked cells after the LICUS irradiation: at the frequency of our experiments (∼1 MHz). This unique behavior occurs despite complex 2D sound pressure patterns being established within the treatment well, with pressure nodes and antinodes separated approximately 375 microns apart in both horizontal *x* and *y* directions shown in Figure 1B. However, the impact of these spatial differences on the collective dynamic behavior of cells in the monolayer was not observed in any of the movies.

### 3.2 Influence of starvation on the wound healing processes after LICU irradiation

Four different types of experiments were carried out according to the combination of two variables or conditions: LICU irradiation/non-irradiation and serum starvation/non-starving conditions for 24 h before the treatment/culture, respectively to study the cell mechano-behavior. Each of these 4 conditions was repeated at least 3 times, so it was necessary to carry out at least 12 experiments. Thus, six assays were performed under starving conditions prior to scratching, to prevent cell proliferation during the cell culture. Three of them included LICUS treatments after the starvation to be compared with the other three samples without ultrasonic irradiation and provided different results.

**FIGURE 4:**
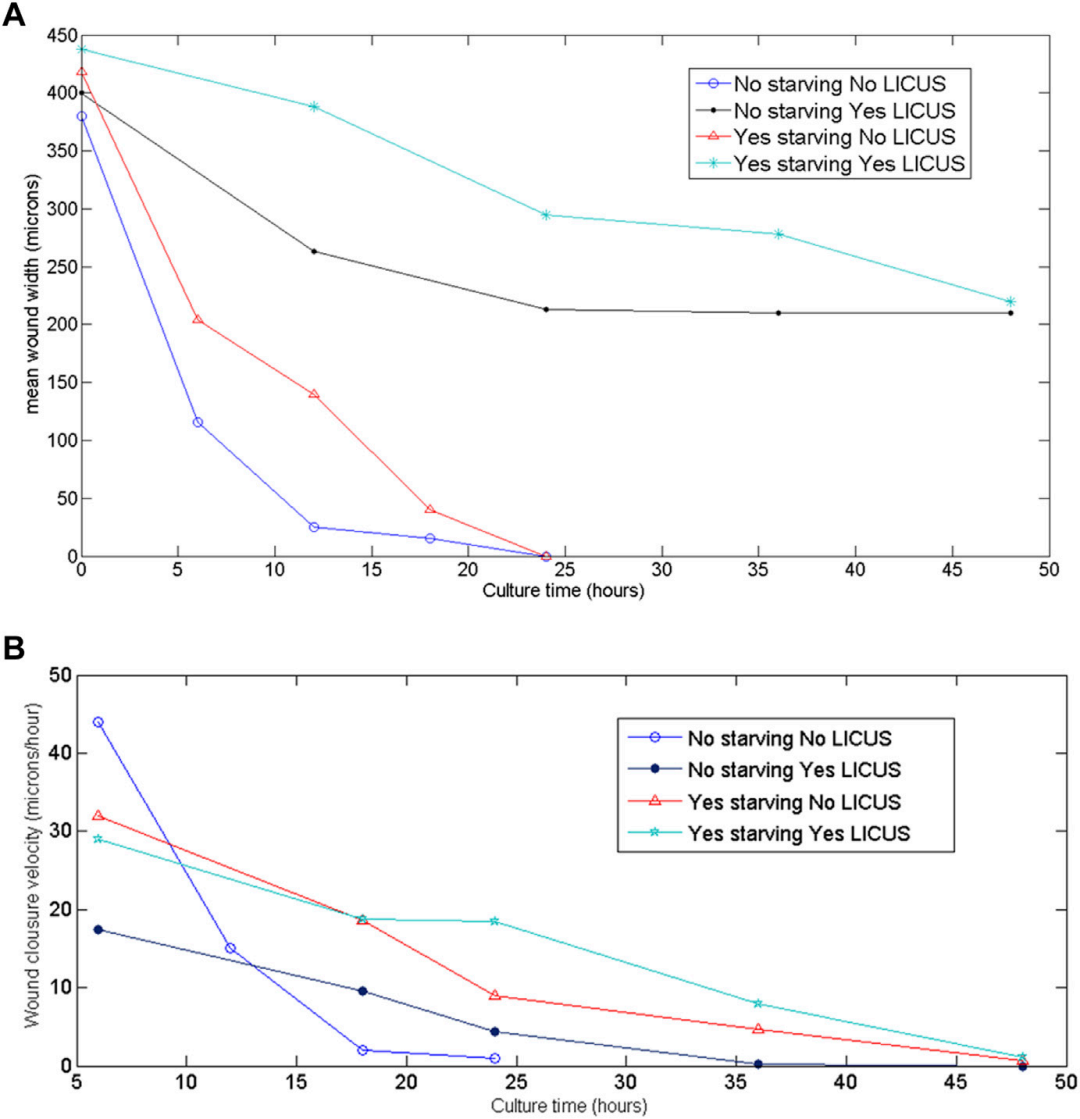
**(A)** Wound widths measured over time on the filmed monolayer samples at different conditions of the experiments; **(B)** quantified wound closure velocities derived from the filmed images of the samples at the different conditions of the experiments.

Quantified results of mean wound width and closure velocity are shown in Figure 4 over time. They were determined from movies taken on starved and not starved samples respectively. Initial wound widths of 400 µm were made on the PANC-1 monolayers to perform the experiments with identical geometrical configurations.

In these figures, blue and black graphics correspond to non-starved samples exposed and not exposed to LICUS treatment at the beginning of the experiment respectively. Red and magenta colored graphics describe wound healing width and velocities over time in starved samples (with and without previous LICUS treatment respectively).

All the curves in this figure decay to zero values: starved samples at the end of the culture process and not starved samples before. This means that they either close fully the gap without ultrasonic irradiation (blue and red lines) or they stop their wound healing process without culminating the gap closure after LICUS actuation (black and magenta lines). At any of both acoustic conditions, starved samples acquire smaller velocity amplitudes over time. The wound width decreases over time during healing processes under normal conditions (without application of external forces) while faced monolayers at both sides of the wound approach each other, showing a decrease on cell velocities until the cell confluence. This happened in both, starved and not starved samples (blue and red graphics of Figures 4A, B). The non-starved samples not previously exposed to LICUS (blue graph) close the wound in the shortest times (before 24 h), with a very rapid decrease in the speed of approach of the wound fronts in an equivalent time, until wound closure.

However, we found less temporal decrease in the wound width in those samples that had been previously LICUS irradiated, both with starvation and without starvation. (black and magenta colored graphics of Figure 3A). In fact, in the experiments the cell confluence of the two fronts of the wound is not reached at least during a 48-h culture. On the contrary, the wound width remains opened for at least 2 days after low intensity ultrasound irradiation. This evidences a long-term inhibition of cell migration in the wound healing process of cancer monolayers.

In summary, non-starved cell monolayers show quantitatively remarkable effects on the cell migration processes from the first hours of culture when irradiated with ultrasound. However, the monolayers subjected to fasting prior to the experiments did not show the effects of LICUs on the collective cell motion so clearly during the first 24 h of cultivation, but rather they were manifested later, on the second day, and they are not as prominent effects as those found in the experiments without starving.

**FIGURE 5:**
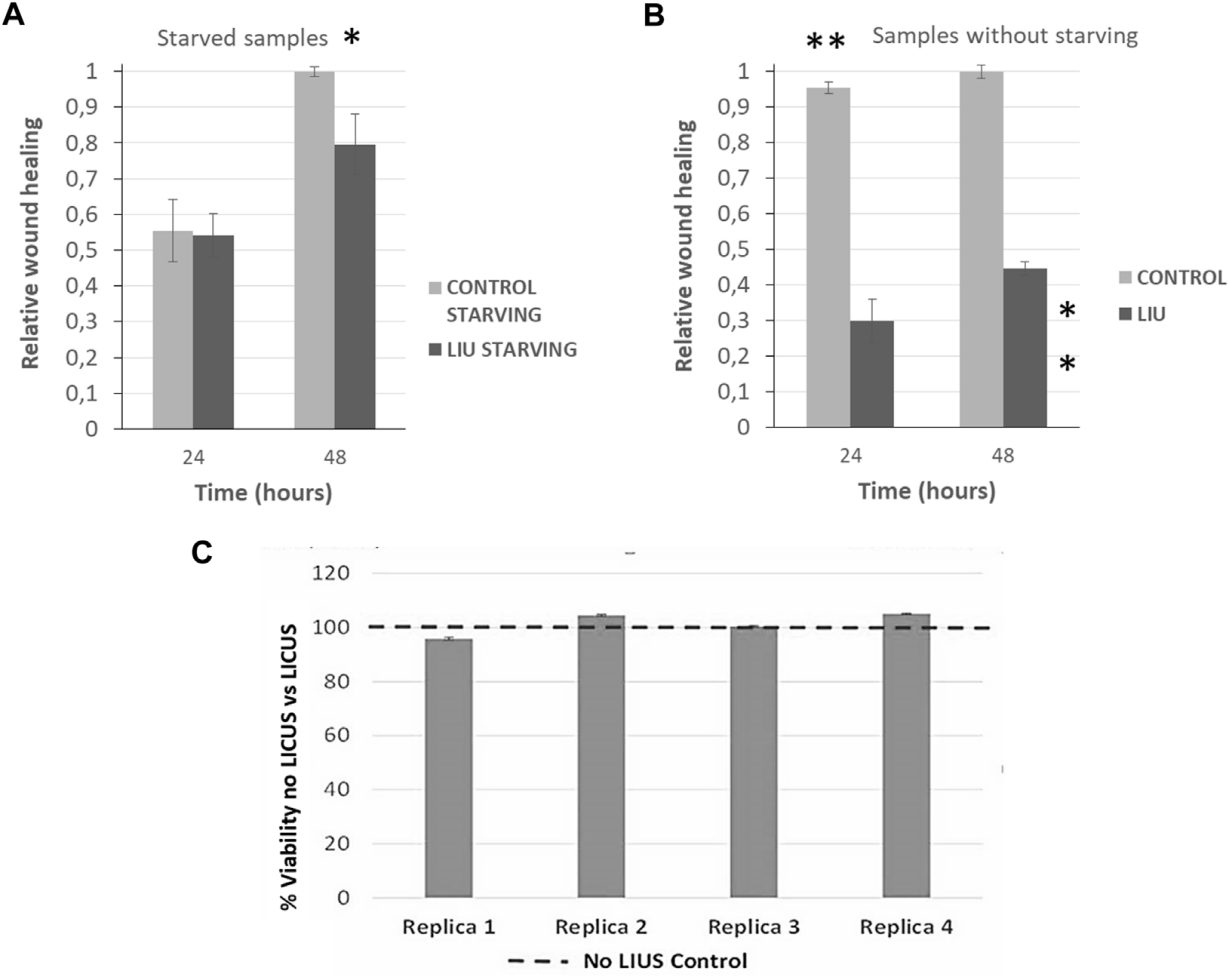
Quantification of relative wound closure of PANC-1 cells in normal conditions (grey bars) and after 20 min of LICU actuation (black bars) after 24 h and 48 h (n = 3)in **(A)** starved monolayers and; **(B)** in non-starving conditions. Statistical significance *p ≤ 0.05, **p ≤ 0,01. *U* Mann-Whitney test; **(C)** Analysis of cell viability after LICUs treatment using Alamar BLUE assays performed in four replica experiments.

Figures 5A, B show quantified results of relative wound healing over time for the four test conditions described above.

### 3.3 Relative wound closure in not starved samples

Monolayers previously exposed to starvation provided a value of 0.5 relative to wound closure after 24 h of culture in normal conditions (black bars corresponding to control samples), as grey bars show in Figure 5A. The effects of LICUS on these samples are negligible during the first day of culture, but become more remarkable in the next 24 h, where the relative wound closure ratio reached 0.2 points lower in treated cells than in control cells (grey bars). After 48 h, these effects became noticeable, reaching a difference of approximately 20% in the relative wound closure on samples exposed to the acoustic irradiation. Furthermore, statistical analysis confirmed the significant difference between the two groups at 48 h, *p ≤ 0.05. As starvation is commonly used to ensure that only migration in wound healing assays is being assessed, this experiment confirms the effect of LICUS on migration inhibition of PANC-1 cancer cells.

### 3.4 Relative wound closure in not starved samples

Wounds of monolayer samples not starved closed much more rapidly than those with starvation in normal conditions without LICUS (grey bars), probably due to a combined effect of cell migration and proliferation. An increase of approximately 0.9 was found after 24 h in control cultures under non-starving conditions. However, after 48 h, the relative wound closure value was similar in both cases (starving and non-starving), showing a long-term response convergence. In addition, a notable influence of LICUs was found on these not starved samples, with relative wound closure at 24 h of culture, (black bar in Figure 4B). Surprisingly, both the relative wound closure and the statistical analysis revealed the significant impact of LICUS on non-fasting samples over time, from the first 24 h of culture after ultrasonic irradiation. The relative wound closure ratio was decreased by 0.6 points in the first 24 h and 0.5 points in the posterior 24 h compared to non-treated cells. This was supported by statistical analysis, which showed a **p ≤ 0.01 in both cases.

### 3.5 Analysis of cell viability

Analysis of cell viability was performed 12 h after the end of the experiments, once finished the culture process of 48 h o 72 h depending on the experiments. Alamar BLUE assays were used to perform this study in four replicate experiments (Figure 5C). This analysis was made on both serum-starved samples and on serum-containing samples with similar viability results.

**FIGURE 6:**
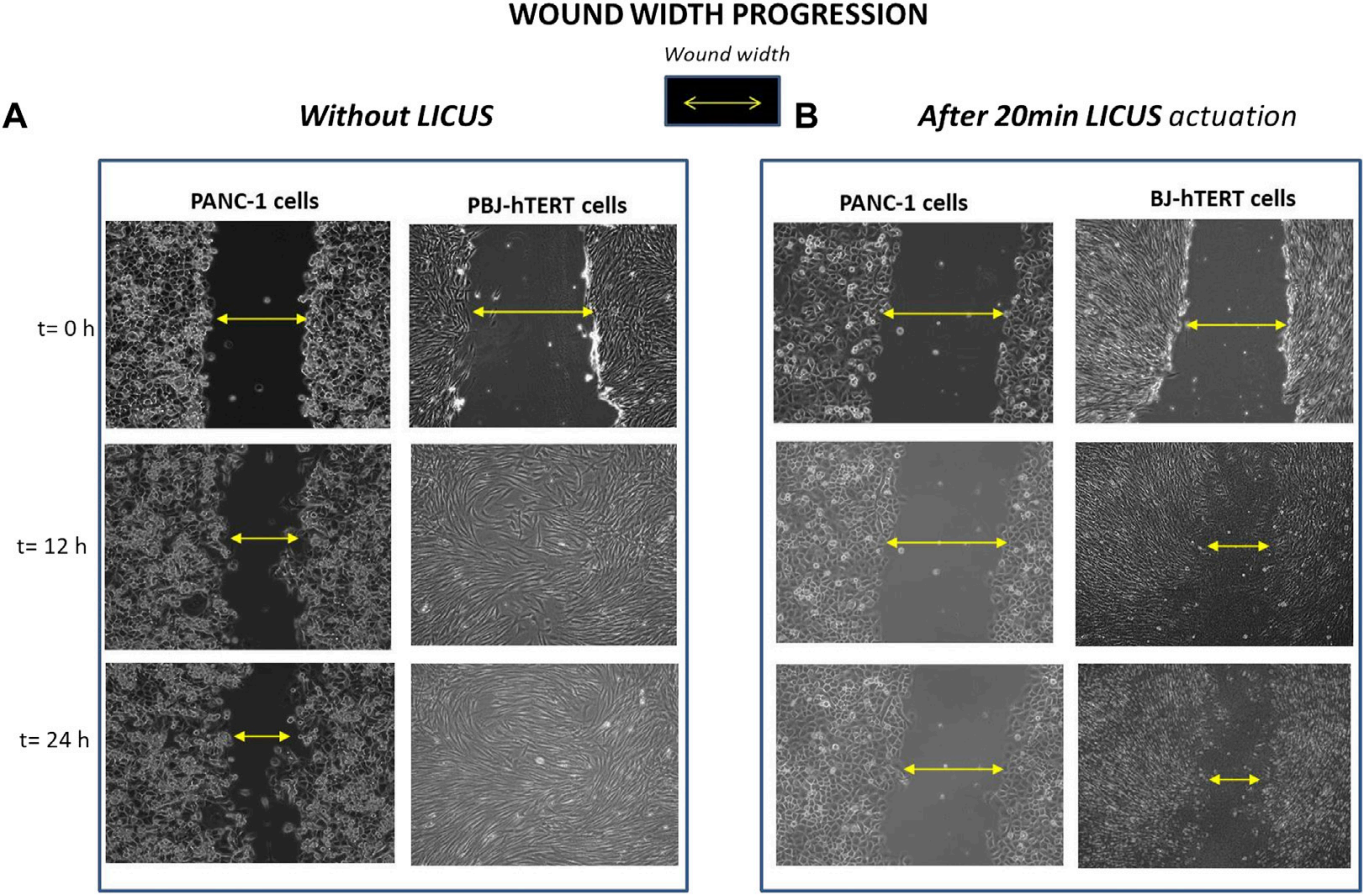
Wound width progression filmed over time in PANC-1 and Fibroblast BJ-hTERT monolayer samples respectively: **(A)** without LICUS exposure before the culture; **(B)** after 20min LICUS irradiation.

Finally, other experiments were performed in the device with fibroblast monolayers to compare the effects of LICUS on the cell migration in both different cell lines, which are displayed in Figure 6 without LICUS irradiation (Figure 6A) and after a exposure to 20min-LICUS respectively (Figure 6B).

Comparison of results of Figure 6 show different influence of the LICUS irradiation on the wound healing processes of PANC-1 and BJ-hTERT respectively performed at the same acoustic and geometrical conditions. Fibroblasts show greater mobility than pancreatic cells, as it can be seen in the filmed frames of Figure 6A, where it can be observed how they reach confluence in 12 h. However, fibroblasts are also susceptible to ultrasonic irradiation, as shown in Figure 6B.

After exposing these cells to irradiation of LICUS for 20 min, the monolayer gap remains partially opened during the first 24 h of culture, in contrast to their behaviour under normal conditions (without acoustic treatment), whose wound closes in approximately 12 h. In contrast, PANC-1 cells have very low mobility, either under normal wound healing conditions or after being irradiated with LICUS. In normal conditions, PANC-1 monolayers require more than 24 h to close a wound with a width similar to that of fibroblast monolayers. Once exposed to the same LICUS dose irradiation, they maintain a wider wound space than fibroblasts at least for 48 h culture.

Other analyses are being performed in the experiments regarding the influence of LICUS on some gene expressions. In particular, a diminution of the vascular endothelial growth factor receptor 3 (VEGFR-3) as well as VEGFR 5-7 and a rise of some interleukins have been found after the LICUS actuation on the samples, which is overexpressed in cancer cells. An in-depth study of these and other changes in gene expression involved in pancreatic cancer processes is required as the next step in this study.

## 4 Discussion

The effects of low-intensity ultrasound on collective cell migration were investigated in more than 20 experiments and are described in this paper. In summary, LICUs hinder migration of epithelial cancer cells in wound healing-induced processes. In particular, we show how pancreatic PANC-1 cancer cells grown in monolayers *in vitro* with a wound, sense and respond to these waves at frequencies close to 1 MHz and low intensities (<100 mW cm^−2^) for 48–72 h after ultrasound irradiation. In particular, the wound remains open for at least 48 h or even 72 h after short-term acoustic treatments of less than 25 min, depending on the ultrasonic dose selected. Despite the strong spatial gradients of acoustic pressure established within the area of the well containing the wound in the monolayer, of few hundreds of microns, the cells behaved as a whole, showing a collective dynamic response to acoustic performance, a bulk-like behavior of the cell monolayers after their exposure. This behavior was also observed in previous experiments carried out on tumor pancreatic explants treated with LICUs for longer times, up to 2 hours of acoustic irradiation (Pajic-Lijakovic and MIlivojevic, 2020).

In addition, the effects of LICUs on samples without starving were more prominent than those of the monolayers previously exposed to starvation for 24 h, showing quantitatively remarkable effects from the first hours of culture, unlike starved monolayers, without apparent LICUS effects during the first 24 h of cultivation.

Low-intensity ultrasound deactivates cell migration processes at certain acoustic conditions, in particular those tested in this research, as demonstrates a paralysis in the wound healing process of cancer cell monolayers during at least 48 h. This work is the first proof-of-concept study to demonstrate the physical effects of ultrasound stimulation on pancreas tumor cell migration. An in-depth biological study of the effects of ultrasounds is on-going but out of the scope of this article. Biochemical and other effects involved in the acoustic response of the cell monolayers are not contemplated as well as mechanisms associated to the role of viscoelasticity in provoking apparent inertial effects and generating the oscillatory mechanical instabilities in the form of standing waves and propagative waves caused by collective cell migration (Health Effects of Exposure to Ultrasound and Infrasound, 2010; Pajic-Lijakovic, 2021). These waves could be affected by the ultrasound actuation if they present similar frequencies and amplitudes of the same order of magnitude of the ultrasonic waves, but would be negligible in other conditions. This latter is the case in our experiments, since these waves correspond to frequencies much lower than those of the applied ultrasonic field.

The findings from this study highlight relevant inhibitory effects of ultrasound on cell migration not reported before and it seems that LICUS could have effects on cell proliferation from the experiments carried out on samples without prior fasting. These findings open a door to the study of the effects of low intensity ultrasound *in-vivo* with tumor tissues that cannot be exposed to fasting.

As outline for a further research, we have found that certain genes directly involved in the cell migration/mobility show variations in their respective expression in the samples previously exposed to LICUS.

A non-invasive technology capable of jointly inhibiting migration and proliferation will be especially relevant in non-invasive slowing down tumor progression.

## Data Availability

The original contributions presented in the study are included in the article/Supplementary Materials, further inquiries can be directed to the corresponding author.
